# *In vivo* high-resolution structural MRI-based atlas of human thalamic nuclei

**DOI:** 10.1038/s41597-021-01062-y

**Published:** 2021-10-28

**Authors:** Manojkumar Saranathan, Charles Iglehart, Martin Monti, Thomas Tourdias, Brian Rutt

**Affiliations:** 1 Department of Medical Imaging, University of Arizona, Tucson, AZ USA; 2 Department of Electrical and Computer Engineering, University of Arizona, Tucson, AZ USA; 3 Department of Psychology, University of California, Los Angeles, CA USA; 4 Service de Neuroimagerie Diagnostique et Thérapeutique, Université de Bordeaux, Bordeaux, France; 5 Department of Radiology, Stanford University, Palo Alto, CA USA

**Keywords:** Brain, Magnetic resonance imaging

## Abstract

Thalamic nuclei play critical roles in regulation of neurological functions like sleep and wakefulness. They are increasingly implicated in neurodegenerative and neurological diseases such as multiple sclerosis and essential tremor. However, segmentation of thalamic nuclei is difficult due to their poor visibility in conventional MRI scans. Sophisticated methods have been proposed which require specialized MRI acquisitions and complex post processing. There are few high spatial resolution (1 mm^3^ or higher) *in vivo* MRI thalamic atlases available currently. The goal of this work is the development of an *in vivo* MRI-based structural thalamic atlas at 0.7 × 0.7 × 0.5 mm resolution based on manual segmentation of 9 healthy subjects using the Morel atlas as a guide. Using data analysis from healthy subjects as well as patients with multiple-sclerosis and essential tremor and at 3T and 7T MRI, we demonstrate the utility of this atlas to provide fast and accurate segmentation of thalamic nuclei when only conventional T_1_ weighted images are available.

## Abstract (summary)

**Table:** 

Measurement(s)	thalamic nuclei
Technology Type(s)	multi-atlas segmentation
Sample Characteristic - Organism	Homo sapiens

Machine-accessible metadata file describing the reported data: 10.6084/m9.figshare.16601045

## Background & Summary

The thalamus has historically been associated with filtering and relaying sensory and motor signals to the cortex. It is also involved in the regulation of sleep, attention, waking, consciousness^1^, and episodic memory^2^. Histologically and functionally, the thalamus is divided into subdivisions called nuclei with specific projections to different cortical areas and associated with specific neurological functions. Thalamic nuclei involvement is increasingly reported in a number of neurodegenerative and psychiatric disorders such as multiple sclerosis^3–5^, alcohol use disorder^6^, schizophrenia^7^, and Parkinson’s disease^8^ among others. Specific nuclei such as the ventralis intermedius nucleus are being targeted for treatment of essential tremor^9^. However, thalamic nuclei are largely invisible on conventional T_1_ or T_2_ weighted MRI sequences. Specialized techniques such as susceptibility weighted imaging^10,11^ have been demonstrated at 7T for delineation of thalamic nuclei, usually involving manual segmentation. These have been used for targeting the ventralis intermedius nucleus for deep brain stimulation surgery^12,13^. Diffusion weighted imaging (DWI) based methods have shown promise for delineation of thalamic nuclei. Local^14,15^ properties such as orientation of the diffusion tensor have been utilized to segment the thalamic nuclei into multiple nuclei at 3T. To date, the most consistent and stable DWI-based technique uses orientation distribution functions represented by a spherical harmonic basis to cluster the thalamic nuclei^16^ at 3T. However, DWI uses echo-planar imaging for its underlying acquisition making it subject to spatial distortion and limiting its spatial resolution. Furthermore, the predominance of grey matter in the thalamus reduces diffusion anisotropy. As a result, DWI-based methods have been successful in only segmenting the larger nuclei.

Over the years, several MRI atlases for thalamic nuclei have been reported. Behrens *et al*.^17,18^ used probabilistic tractography to create an atlas with seven sub-regions. However, this atlas is based on structural connectivity to the cortex rather than anatomical correspondence to a histological atlas. While the Krauth atlas^19^ is a digital representation of the Morel stereotactic atlas^20^, it is built using 3 healthy *postmortem* brains. The probabilistic atlas of Iglesias *et al*.^21^ is also, primarily, based on 6 *postmortem* brains. Recently Najdenovska *et al*.^22^ reported an atlas based on the DWI clustering method of Battistella *et al*.^16^ using 70 healthy subjects from the Human Connectome Project. This atlas had seven clusters, six of which loosely corresponded to larger thalamic nuclei while the seventh cluster was a conglomerate of three histologically-defined nuclei. Resting state functional MRI based methods have also been used to segment thalamic nuclei and create atlases by Zhang *et al*.^23^ and Kumar *et al*.^24^. Even though qualitative correspondence to the Morel atlas was noted, there were no direct quantitative comparisons to manual segmentation ground truth in most of these methods.

T_1_- and T_2_-weighted structural MRI is usually performed at much higher spatial resolution than EPI-based methods which underlie DWI and functional MRI and would be more suitable for high spatial resolution atlas creation. However, T_1_ weighted Magnetization Prepared Rapid Gradient Echo (MP-RAGE) or T_2_ weighted fast spin echo structural imaging sequences possess very little inter-nuclear contrast to be of value in nuclei segmentation. Liu *et al*.^25^. used a combination of susceptibility weighted and MP-RAGE data acquired with different contrasts at 7T to create a manual segmentation multi-atlas which was then used to segment 3T MP-RAGE data. Recently, a method for thalamic segmentation called Thalamus optimized multi atlas segmentation (THOMAS)^26^ based on a variant of MP-RAGE has been proposed for 7T which shows great promise for high resolution thalamic nuclei segmentation. However, THOMAS requires the acquisition of a white-matter-nulled (WMn) MP-RAGE sequence^27,28^ to improve intra-thalamic contrast, which has not generally been part of the suite of standard MRI sequences. This also prevents retrospective analysis of large databases like Alzheimer’s Disease Neuroimaging Initiative (ADNI), which include only conventional structural imaging sequences like MP-RAGE.

The goal of this work was to create a high spatial resolution (0.7 × 0.7 × 0.5 mm^3^) *in vivo* MRI structural atlas based on a database of WMn MP-RAGE data, which were segmented manually using the Morel stereotactic atlas as a guide. This allowed delineation of thalamic nuclei from conventional MP-RAGE, enabling their segmentation from existing standard clinical imaging protocols. We describe the creation of this atlas and demonstrate its utility using 3T and 7T MRI data sets.

## Methods

### Datasets and manual segmentation

The structural atlas proposed in this work was generated using 9 WMn MP-RAGE prior datasets (6 male, 3 female; age = 24–43 years, mean = 32 years, SD = 5 years) acquired on a GE 7T MRI system from healthy control subjects with the following parameters:

180 coronal slices, TR/TE 6,000/10 ms, inversion time 680 ms, flip angle 4°, 0.7 × 0.7 × 0.5 mm^3^ image resolution, FOV 180 mm, parallel imaging factor 1.5 × 1.5 (6 datasets with no parallel imaging).

**Fig. 1:**
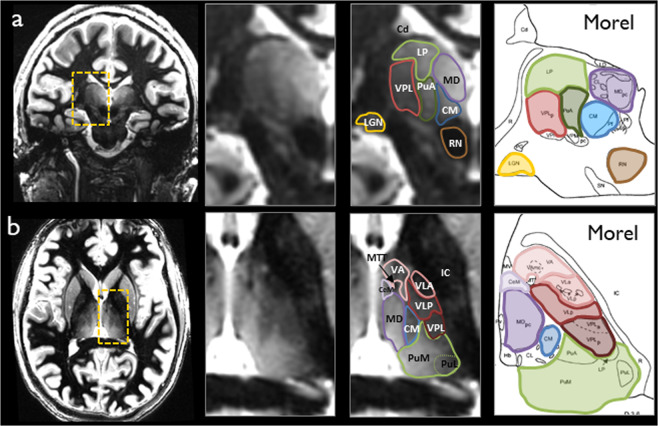
Two example WMn-MPRAGE data sets in coronal (**a**) and axial (**b**) planes with manual segmentation overlays along with the approximate slice location in the Morel atlas for reference.

While histology is considered the gold standard, manual segmentation performed by an expert neuroradiologist using the Morel atlas^20^ as a reference was used in this work involving *in vivo* data where histological data are not available. A reproducible manual segmentation protocol was developed with excellent intra-rater reliability as measured by intraclass correlation coefficient (ICC) and mean distance discrepancy between centers of mass (ΔCoMs) for initial and repeat tracings 3 weeks later, yielding ICC of 0.997 (95% confidence interval 0.996–0.998) and ΔCoM of 0.69 ± 0.38 mm respectively. More details of the manual segmentation procedure can be found in Tourdias *et al*.^27^ Two example data sets in axial and coronal planes are shown in Fig. 1, demonstrating the superior contrast of the WMn MP-RAGE sequence that enables clear demarcation of nuclei boundaries for manual delineation in conjunction with the Morel atlas. All the prior datasets were manually segmented to identify 11 thalamic nuclei and the mammillothalamic tract (MTT). The eleven delineated nuclei are grouped as follows:

(i) **anterior group**: anteroventral (AV)
(ii) **lateral group**: ventral posterolateral (VPL), ventral lateral anterior (VLa), ventral lateral posterior (VLp), ventral anterior nucleus (VA)
(iii) **medial group**: mediodorsal (MD), centromedian (CM), habenula (Hb)
(iv) **posterior group**: pulvinar (Pul), medial geniculate nucleus (MGN), lateral geniculate nucleus (LGN)

### Custom template construction and atlas creation

**Fig. 2:**
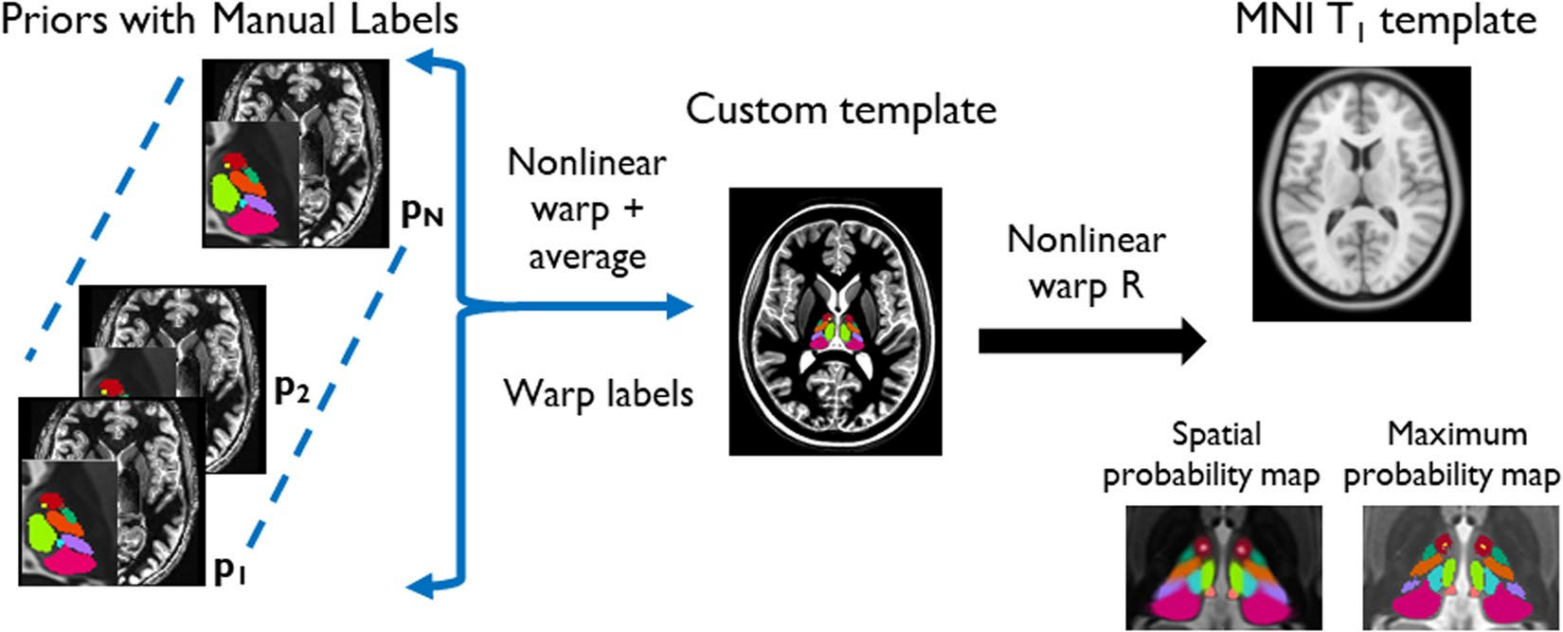
Main steps in the creation of the proposed thalamic atlas.

A custom template was created using the *buildtemplate* script of Advanced Normalization Tools (ANTs^29^) as described in Su *et al*.^26^. Briefly, this is achieved by iteratively registering each of the 9 priors to the average of the priors and then averaging the registered priors to create a custom template which has very high signal-to-noise ratio and contrast whilst including normal and diseased brain states. Registration was first affine followed by nonlinear warping using the symmetric group-wise diffeomorphic normalization (SyN) algorithm of ANTs. ANTs was chosen for its accuracy and precision as reported by Klein *et al*.^30^. The nonlinear warps from each prior to the custom template were also computed using ANTs. Finally, labels were transferred from the space of the 9 priors to the custom template space using the warps computed above and nearest-neighbor interpolation to generate the thalamic parcellations in template space. These 9 parcellations were then combined to calculate the spatial probability maps and maximum probability map using custom python scripts. Spatial probability maps were generated by computing the relative frequencies of labels at each voxel in template space to yield the probability of that voxel belonging to each thalamic nucleus. Maximum probability maps were computed using the mode of these distributions, thus assigning a single label to each voxel representative of the most probable thalamic nucleus at that location. All the final maps are at 0.7 × 0.7 × 0.5 mm spatial resolution. Lastly, the custom template was nonlinearly registered to MNI space (nonlinear ICBM152 asymmetric^31^) and this spatial warp was saved and used to warp the probability maps from custom template to MNI space. Transformations from the priors to custom template and custom template to MNI space were concatenated to minimize interpolation errors during generation of the thalamic nuclei labels in MNI space with 0.5 mm isotropic spatial resolution. These steps are summarized in Fig. 2.

## Data Records

**Fig. 3:**
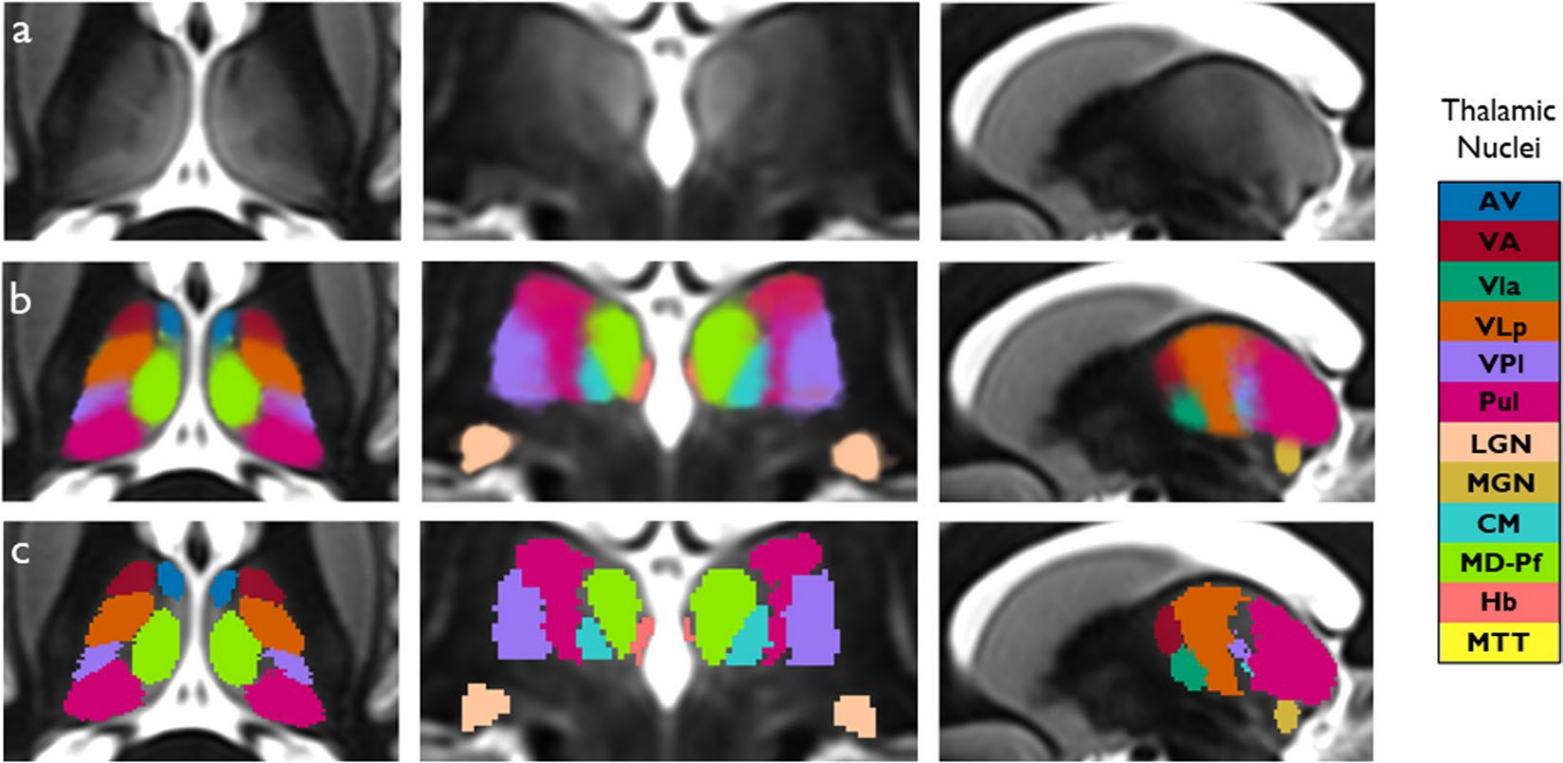
Spatial probability maps (**b**) and maximum probability maps (**c**) in custom WMn MP-RAGE template space shown in three orthogonal planes. The top row shows the custom WMn MP-RAGE template without overlays for reference.

**Fig. 4:**
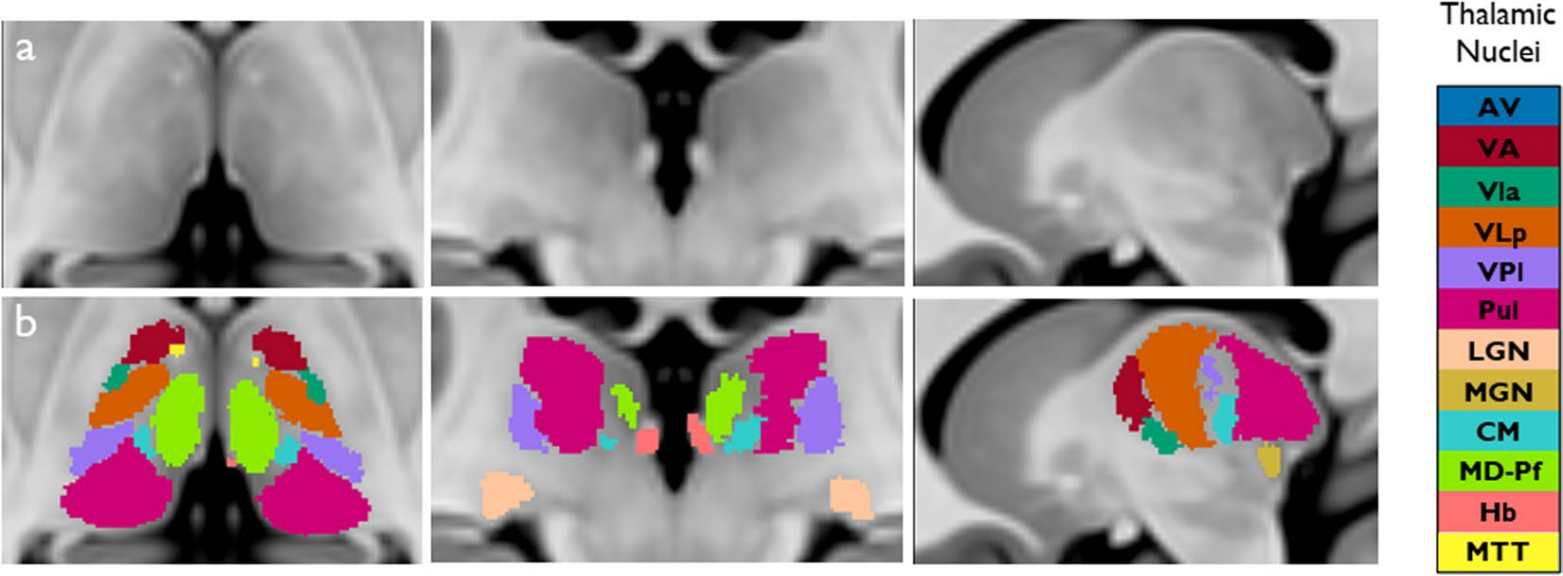
Maximum probability thalamic maps overlaid (**b**) in MNI 152 space (**a**) in three orthogonal planes.

The primary contribution of our work are the spatial probability and maximum probability maps of thalamic nuclei in custom template space at 0.7 × 0.7 × 0.5 mm resolution. They are in compressed NIfTI-1 format (i.e. .*nii.gz* extension) with the spatial probability map a 4-D file, the 4^th^ dimension of size 24 for the 12 left and 12 right thalamic nuclei and separate maximum probability maps for left and right thalami. The maximum probability maps are also provided in MNI 152 (nonlinear 2009b^31^) space at 0.5 mm isotropic resolution. In addition, the inferior half of the VLp nucleus i.e. the ventralis intermedius (VIM) nucleus is also provided as a separate file in both custom template and MNI spaces for the left and right sides. All file names follow the BIDS naming convention. Figure 3 shows the spatial probability maps and maximum probability maps overlaid on the custom template in all three planes. The maximum probability map in MNI space is shown in Fig. 4.

A customized color lookup table (*.ctbl* extension) recognized by standard visualization tools like 3D Slicer is also provided. This can be custom edited to change the color scheme or add additional nuclei. The data are available through zenodo^32^. In addition, code for segmentation of conventional MP-RAGE using the atlas and some extra files used by the code are also provided (see Code availability section). The original WMn MP-RAGE datasets used for atlas creation and their segmentation are available through zenodo^33^ in compressed NIfTI-1 format (i.e. *.nii.gz* extension). A summary of data records is shown in Table 1.

**Table 1:** Summary of data records related to this study.

Dataset	Data for atlas creation	Data used for testing
Number of subjects	9	36
Provenance	Prior data from Su *et al.*^19^	Mixed (see Methods)
Available from	Zenodo^33^	Zenodo Subset^32^
Modalities used	WMn MP-RAGE	WMn MP-RAGE and MP- RAGE
Use	Atlas creation	Atlas validation
Provided output	Spatial and max. probability maps	THOMAS and atlas-based thalamic nuclei
Output format	NifTI-1 (.gz) 4D and 3D BIDS naming convention	NifTI-1 (.gz) 3D

## Technical Validation

To validate the accuracy of the nonlinear registration from the custom template to the MNI template, six anatomical landmarks were placed by an expert neuroradiologist on the MNI template and the custom template warped to MNI space and the distance computed between the two for each landmark, which represents warp errors arising from the nonlinear registration step. The six landmarks included anterior and posterior commissure, left and right mammillary bodies, left and right habenula, left and right peak of the pulvinar nucleus, and the left and right mammillothalamic tract (Supplemental Fig. 1), covering areas adjacent to and within the thalami.

To test the accuracy of the proposed atlas-based segmentation method, two datasets were used. The first comprised of data from 18 subjects- 13 patients with essential tremor, 4 with multiple sclerosis, and one healthy subject (9 male, 9 female; age = 41-86 years, mean = 67.8 years, SD = 14.5 years) acquired on a 7T GE scanner using a 32-channel array (Nova Medical Systems). These were completely separate from the prior subjects used for atlas construction. The second test dataset comprised of 18 healthy subjects (15 male, 3 female; age = 18-44 years, mean = 25.3 years, SD = 7.8 years) acquired on a 3T Siemens Prisma scanner using a 32-channel array. All subjects were scanned after written informed consent adhering to institutional review board (IRB) guidelines. The scan parameters for the sequences were as follows:

**7T**: *Conventional MP-RAGE*- 180 coronal slices, TR/TE 3,000/7.2 ms, flip angle 6°, inversion time 1200 ms, 1 mm isotropic resolution, Field of view (FOV) 180 mm, Autocalibrating reconstruction for Cartesian imaging (ARC) acceleration factor 2.

*WMn MP-RAGE*- 180 coronal slices, TR/TE 6,000/10 ms, inversion time 680 ms, flip angle 4°, 1 mm isotropic resolution, FOV 180 mm, ARC factor 1.5 × 1.5.

**3T**: *Conventional MP-RAGE*- 192 sagittal slices, TR/TE 2,000/2.52 ms, flip angle 12°, 1 mm isotropic resolution, FOV 256 mm, generalized autocalibrating partially parallel acquisitions (GRAPPA) factor 2.

*WMn MP-RAGE*: 160 axial slices, TR/TE 4,000/3.75 ms, inversion time 500 ms, flip angle 7°, 1 mm isotropic resolution, FOV 256 mm, GRAPPA factor 2.

To perform thalamic nuclei segmentation on conventional MP-RAGE data using the proposed atlas, the input images were first bias corrected using the N4 bias correction function of ANTs, then automatically cropped to encompass bilateral thalami and finally nonlinearly registered to a cropped custom template using Mutual Information (MI) for the registration cost function. The use of N4 bias correction and cropping to cover both thalami ensured minimal distortion and B_1_ inhomogeneity effects even on 7T MRI data. The custom template thalamic nuclei labels were than warped back to input space using nearest-neighbor interpolation. A simple shell script for performing this segmentation is also included in the distribution.

For both 7T and 3T datasets, WMn MP-RAGE images from each patient were segmented using THOMAS and conventional MP-RAGE using the proposed atlas-based segmentation approach, respectively.

**Fig. 5:**
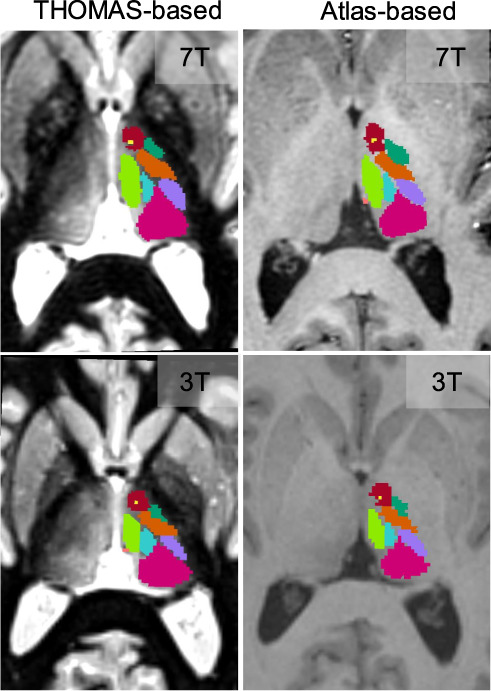
Comparison of THOMAS-based and the proposed atlas-based segmentation on an MS patient at 7T (top row) and a healthy subject at 3T (bottom row).

For the 7T data, manual segmentation on WMn MP-RAGE performed by a trained neuroradiologist guided by the Morel atlas were also available. As a result, THOMAS and the atlas-based segmentations were individually compared to the manual segmentation ground truth. For the 3T data set, the atlas-based segmentation was directly compared to THOMAS segmentation, due to lack of manual segmentation ground truth. The WMn and conventional MP-RAGE data from each patient were affine registered to each other prior to quantitative comparisons. Their associated labels were also registered by applying the same affine transform with nearest neighbor interpolation. Figure 5 shows comparison of THOMAS segmentation on WMn MP-RAGE (left column) with atlas-based segmentation on conventional MP-RAGE (right column) for a MS patient at 7T (top row) and a healthy subject at 3T (bottom row). The qualitative agreement of the methods can be appreciated.

The main quantitative measures used for comparisons were Dice coefficients and Volume Similarity Index (VSI). These are defined as

1 $${\rm{Dice}}=2\frac{\left|A\cap B\right|}{\left|A\right|+\left|B\right|}\;{\rm{and}}\;{\rm{VSI}}=1-\frac{abs\left(\left|A\right|-\left|B\right|\right)}{\left|A\right|+\left|B\right|}$$

where A and B refer to the two segmentation labels compared and |A| and |B| refers to the number of pixels in A and B respectively.

### Results

Misregistration errors between the landmarks in MNI space and the custom template warped to MNI space are reported in mm in Table 2 and attest to the quality of the nonlinear registration step.

**Table 2:** Errors on anatomical landmarks arising from custom template to MNI registration step.

Landmark	Error (mm)
Anterior commissure	0.37
Posterior commissure	0.24
Mammillary body (left)	0.32
Mammillary body (right)	0.35
Habenula (left)	0.26
Habenula (right)	0.34
Pulvinar peak (left)	0.33
Pulvinar peak (right)	0.39
Mammillothalamic tract (left)	0.37
Mammillothalamic tract (right)	0.22

Dice and VSI values for the 7T test data are shown in Table 3 for the whole thalamus and 11 segmented nuclei. For THOMAS segmentation, mean Dice and VSI were 0.73 and 0.91 for the larger nuclei (nuclei 2-6) and 0.64 and 0.86 for the smaller nuclei (nuclei 7-12) using THOMAS. For the atlas-based segmentation, mean Dice and VSI were 0.67 and 0.91 for the larger nuclei and 0.59 and 0.89 for the smaller nuclei. While there is a slight reduction in Dice for the atlas-based method, especially for the smaller nuclei, the reductions are <  = 10% except for VPL (22%) and MGN (11%). VSI was comparable for most nuclei. The mean Dice for all nuclei was 0.68 for THOMAS and 0.63 for the atlas-based segmentation. These are slightly smaller than mean Dice of 0.78 using the shape-based segmentation of Liu *et al*.^23^. and much better than the mean Dice of 0.49 using the registration-based image enhancement method of Bao *et al*.^34^. The THOMAS Dice results are slightly lower than the original THOMAS^26^ results for the smaller nuclei, presumably due to the use of data from patients with essential tremor (13 out of 18) in this work with the attendant motion artifacts. Note that Liu *et al*.^23^. report results only on 9 healthy subjects and do not report small nuclei such as LGN and MGN.

**Table 3:** Dice and VSI values for 7T test data.

Nucleus	Dice THOMAS vs. manual	Dice Atlas vs. manual	VSI THOMAS vs. manual	VSI Atlas vs. manual
*1 Whole thalamus*	0.89 ± 0.02^†^	0.88 ± 0.02	0.95 ± 0.03	0.96 ± 0.03
*2 Pulvinar (Pul)*	0.84 ± 0.03^†^	0.79 ± 0.04	0.95 ± 0.03	0.92 ± 0.06
*3 Ventrolateral posterior (VLp)*	0.76 ± 0.03^†^	0.71 ± 0.05	0.89 ± 0.07	0.89 ± 0.07
*4 Mediodorsal (MD)*	0.83 ± 0.03^†^	0.80 ± 0.04	0.92 ± 0.05	0.93 ± 0.05
*5 Ventral posterior lateral (VPl)*	0.61 ± 0.14^†^	0.47 ± 0.15	0.89 ± 0.09	0.89 ± 0.07
*6 Ventral Anterior (VA)*	0.63 ± 0.09	0.60 ± 0.11	0.92 ± 0.05	0.91 ± 0.08
*7 Anteroventral (AV)*	0.67 ± 0.11	0.60 ± 0.07	0.79 ± 0.13	0.88 ± 0.09
*8 Centromedian (CM)*	0.66 ± 0.14^†^	0.60 ± 0.15	0.91 ± 0.07	0.89 ± 0.08
*9 Lateral geniculate nucleus (LGN)*	0.59 ± 0.15^†^	0.54 ± 0.11	0.89 ± 0.10	0.92 ± 0.07
*10 Ventral lateral anterior (VLa)*	0.52 ± 0.13	0.52 ± 0.11	0.76 ± 0.17	0.81 ± 0.11
*11 Medial geniculate nucleus(MGN)*	0.66 ± 0.10^†^	0.59 ± 0.08	0.87 ± 0.09	0.91 ± 0.08
*12 Habenula (Hb)*	0.74 ± 0.07	0.69 ± 0.06	0.93 ± 0.04	0.94 ± 0.05

^†^indicates p < 0.05/12 for paired t-test comparisons with correction for multiple comparisons (12).

Dice and VSI for the 3T test data are shown in Table 4 for the whole thalamus and 11 segmented nuclei. Note that these are in comparison to THOMAS as opposed to a manual segmentation gold standard. The mean Dice and VSI were 0.8 and 0.95 for the larger nuclei (nuclei 2-6) and 0.7 and 0.91 for the smaller nuclei (nuclei 7-12), indicating a fairly high degree of concordance.

**Table 4:** Dice and VSI values for 3T test data.

Nucleus	Dice Atlas vs. THOMAS	VSI Atlas vs. THOMAS
*1 Whole thalamus*	0.91 ± 0.01	0.98 ± 0.01
*2 Pulvinar (Pul)*	0.85 ± 0.02	0.96 ± 0.03
*3 Ventrolateral posterior (VLp)*	0.80 ± 0.04	0.96 ± 0.02
*4 Mediodorsal (MD)*	0.85 ± 0.03	0.96 ± 0.03
*5 Ventral posterior lateral (VPl)*	0.69 ± 0.08	0.89 ± 0.06
*6 Ventral Anterior (VA)*	0.74 ± 0.05	0.96 ± 0.03
*7 Anteroventral (AV)*	0.72 ± 0.05	0.92 ± 0.10
*8 Centromedian (CM)*	0.74 ± 0.07	0.96 ± 0.04
*9 Lateral geniculate nucleus (LGN)*	0.70 ± 0.07	0.94 ± 0.05
*10 Ventral lateral anterior (VLa)*	0.63 ± 0.10	0.83 ± 0.10
*11 Medial geniculate nucleus (MGN)*	0.73 ± 0.06	0.89 ± 0.06
*12 Habenula (Hb)*	0.71 ± 0.07	0.91 ± 0.05

## Usage Notes

The atlases provided are in slice correspondence with the standard MNI 152 nonlinear 2009b atlases. Code is also provided for users to efficiently derive thalamic parcellation of their input data using the supplied templates and atlases. A readme file explains the different files and their roles.

## Supplementary information

Supplemental Figure 1

## Data Availability

The code for the segmentation is a shell script which is provided in the Zenodo repository^32^. It performs an automatic cropping of the input dataset prior to registering to a cropped custom template. This is done to speed up registration and for accuracy by focusing on the thalami as the crop region encompasses both thalami. A mask for automatic cropping and the cropped custom template are also provided.
