# Supplementary material for: In vivo high-resolution structural MRI-based atlas of human thalamic nuclei
Source: Sci Data. 2021 Oct 28;8:275. doi: 10.1038/s41597-021-01062-y (PMC8553748; doi:10.1038/s41597-021-01062-y)
Supplement: Supplementary file 1 — Supplemental Figure 1 [file 41597_2021_1062_MOESM1_ESM.pdf]

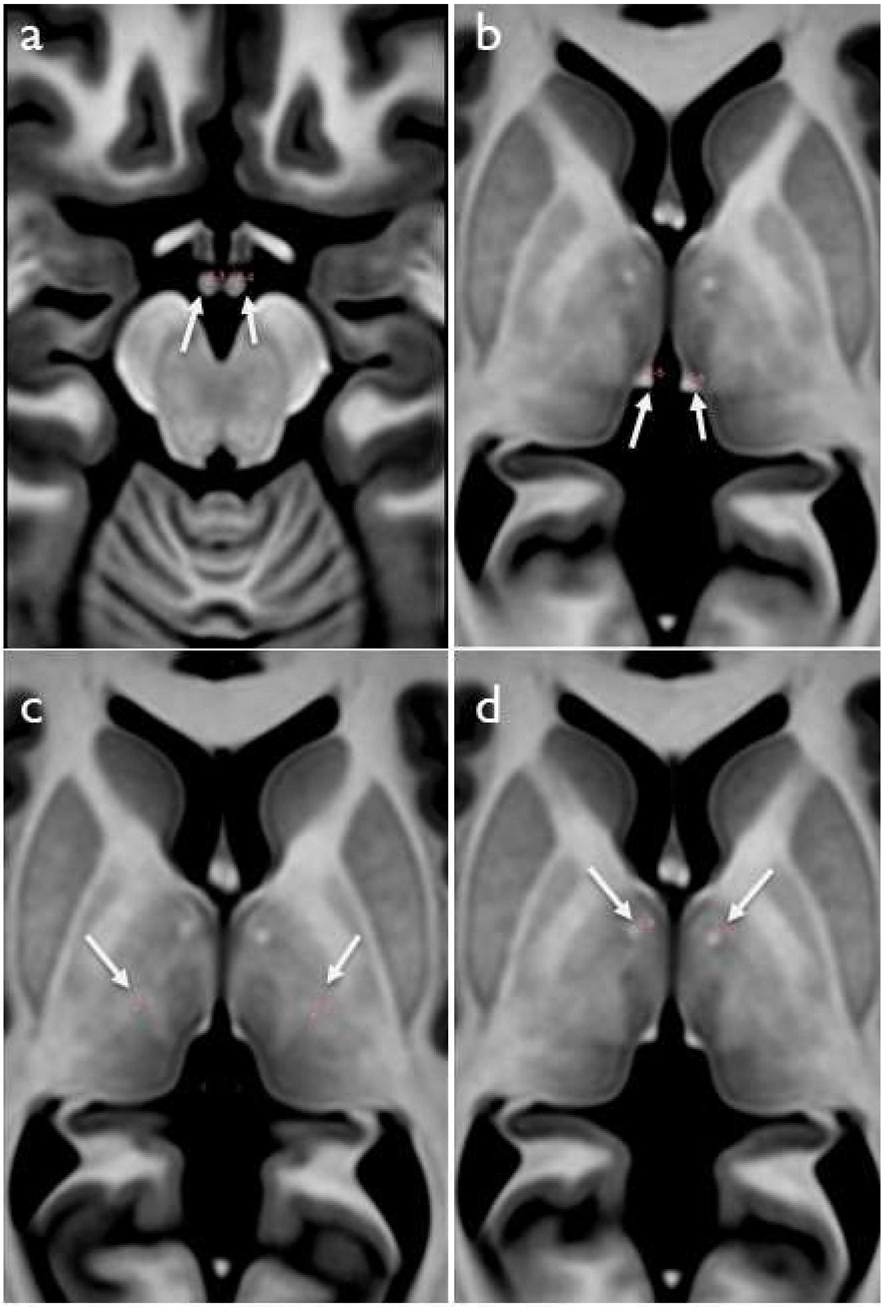

L

Supplementary Figure 1. Anatomical landmarks used to validate the custom template to MNI nonlinear registration- mammillary body (a), left and right habenula (b), left and right pulvinar peak (c), and left and right mammillothalamic tract (d). The anterior and posterior commissure were also used but are omitted here for brevity.
